# Identification of pivotal genes and regulatory networks associated with atherosclerotic carotid artery stenosis based on comprehensive bioinformatics analysis and machine learning

**DOI:** 10.3389/fphar.2024.1364160

**Published:** 2024-04-17

**Authors:** Xiaohong Qin, Rui Ding, Haoran Lu, Wenfei Zhang, Shanshan Wei, Baowei Ji, Rongxin Geng, Liquan Wu, Zhibiao Chen

**Affiliations:** ^1^ Department of Neurosurgery, Renmin Hospital of Wuhan University, Wuhan, Hubei, China; ^2^ Central Laboratory, Renmin Hospital of Wuhan University, Wuhan, Hubei, China; ^3^ Department of Oncology, Wuchang Hospital Affiliated to Wuhan University of Science and Technology, Wuhan, China

**Keywords:** carotid artery stenosis, atherosclerosis, machine learning, pathogenic markers, therapeutic targets

## Abstract

**Objective:**

Bioinformatics methods were applied to investigate the pivotal genes and regulatory networks associated with atherosclerotic carotid artery stenosis (ACAS) and provide new insights for the treatment of this disease.

**Methods:**

The study utilized five ACAS datasets (GSE100927, GSE11782, GESE28829, GSE41571, and GSE43292) downloaded from the NCBI GEO database. The first four datasets were combined as the training set (*n* = 99), while GSE43292 (*n* = 64) was used as the validation set. Difference analysis and functional enrichment analysis were then performed on the training set. The pathogenic targets of ACAS were screened by protein-protein interaction networks and MCODE analyses, combined with three machine learning algorithms. The results were next verified by analysis of inter-group differences and ROC curve analysis. Next, immune-related function and immune cell correlation analyses were performed, and plaques of human ACAS were applied to verify the results via immunohistochemistry (IH) and immunofluorescence (IF). Finally, the competing endogenous RNAs (ceRNA) and transcription factors (TFs) regulatory networks of the characterized genes were constructed.

**Results:**

A total of 177 differentially expressed genes were identified, including 67 genes downregulated and 110 genes upregulated. Gene set enrichment analysis revealed that five pathways were active in the experimental group, including xenograft rejection, autoimmune thyroid disease, graft-versus-host disease, leishmaniasis infection, and lysosomes. Four key genes were identified, with C3AR1 being upregulated and FBLN5, PPP1R12A, and TPM1 being downregulated. The analysis of inter-group differences demonstrated that the four characterized genes were differentially expressed in both the control and experimental groups. The ROC analysis showed that they had high AUC values in both the training and validation sets. Therefore, a predictive ACAS patient nomogram model based on the screened genes was established. Correlation analysis revealed a positive correlation between C3AR1 expression and neutrophils, which was further validated in IH and IF. One or multiple lncRNAs may compete with the characterized genes for binding miRNAs. Additionally, each characterized gene interacts with multiple TFs.

**Conclusion:**

Four pivotal genes were screened, and relevant ceRNA and TFs were predicted. These molecules may exert a crucial role in ACAS and serve as potential biomarkers and therapeutic targets.

## 1 Introduction

As of 2019, stroke remains the second leading cause of death worldwide and the third leading cause of death and disability (Feigin et al., 2021). Ischemic stroke accounts for 87% of these cases (Saini et al., 2021). The primary cause of ischemic stroke is ischemia and even necrosis of brain tissue due to carotid artery stenosis (CAS), occlusion, or detachment of carotid plaque (Feske, 2021). ACAS is a narrowing of the carotid artery diameter due to the formation of carotid atherosclerotic plaques, which is very common, affecting one in five patients with stroke or transient ischemic attack (TIA), and occurs mostly in the bifurcation of the common carotid artery and the beginning of the internal carotid artery (Cheng et al., 2019; Heck and Jost, 2021). Some stenotic lesions may even progress to complete occlusion, resulting in severe neurological deficits, such as coma, limb paralysis, speech disorders, sensory deficits, hemianopsia, intellectual disability, and infarctions in certain areas, such as the brainstem, may even result in sudden death (Kappelle, 2002; Campbell et al., 2019). Treatment options depend on the degree of CAS and the patient’s symptoms, and include medical, surgical, or interventional therapy. Conservative medical treatment aims to reduce the symptoms of cerebral ischemia and lower the risk of stroke; controlling existing diseases such as hypertension, diabetes mellitus, hyperlipidemia and coronary heart disease is the main strategy (Bonati et al., 2022). The aim of surgical treatment is to prevent the onset of stroke, followed by prevention and slowing of the onset of TIA. The standard surgical procedure is carotid endarterectomy (CEA), but CEA also carries potential risks of stroke, heart attack, and hyperperfusion syndrome (Bonati et al., 2022). Carotid angioplasty and stenting is an alternative to CEA, especially in cases where the neck anatomy is not conducive to surgery (White et al., 2022). It is a minimally invasive procedure in which a stent is placed into the carotid arteries to increase blood flow, but there are still problems with intraprocedural endothelial tearing, postprocedural elastic regression of the vessel, and restenosis (Bonati et al., 2022; White et al., 2022). In conclusion, each of the three treatments has its own set of advantages, disadvantages, and indications. With the advancements in vascular imaging technology, the prevalence of ACAS is gradually increasing, how to block or reverse the process of carotid atherosclerotic plaque formation at an early stage and improve the ACAS is the hot spot of current research. Therefore, an in-depth and comprehensive investigation of the causes of carotid atherosclerotic plaque formation and related pathogenic factors is urgently required.

In recent years, machine learning (ML) has been continuously applied to clinical diseases for disease diagnosis, target screening, patient prognosis prediction, and therapeutic programmes due to its powerful computational power, lower error rate and better predictive performance (Swanson et al., 2023; Theodosiou and Read, 2023). In this study, protein-protein interaction networks (PPI) and molecular complex detection (MCODE) analyses were combined with three ML algorithms, namely least absolute shrinkage and selection operator (LASSO), support vector machine-recursive feature elimination (SVM-RFE) and random forest (RF), to screen out critical targets of ACAS, which can offer a new theoretical reference for precise therapy of the illness.

## 2 Materials and methods

### 2.1 Retrieval and merging of datasets

We obtained five datasets from the NCBI GEO database (https://www.ncbi.nlm.nih.gov/geo/): GSE100927 (12 controls + 29 carotid atherosclerosis), GSE11782 (9 controls + 9 carotid atherosclerosis), GESE28829 (13 controls + 16 carotid atherosclerosis), GSE41571 (6 controls + 5 carotid atherosclerosis), and GSE43292 (32 controls + 32 carotid atherosclerosis). The first four datasets were combined as the training set (*n* = 99), while GSE43292 (*n* = 64) was used as the validation set. We then applied the sva package for batch calibration and visualized the pre- and post-correction results using principal component analysis (PCA).

### 2.2 Patients and samples with ACAS

Twenty patients diagnosed with ACAS and admitted to Renmin Hospital of Wuhan University between 2021 and 2023 were included in the study. The control group consisted of 10 ACAS patients who underwent CEA, and the experimental group consisted of 10 ACAS patients who also underwent CEA. The study collected neighboring intima around atherosclerotic plaques in the control group and atherosclerotic plaques in the experimental group, resulting in a total of 20 cases. The atherosclerotic plaques and adjacent intima were collected within 10 min of CEA and stored at −80°C for future use. The study protocol was approved by the Clinical Research Ethics Committee of Renmin Hospital of Wuhan University (Ethics Approval No. WDRY2023-K123), and all methods used complied with relevant guidelines and regulations. Informed consent forms were signed by all participants.

### 2.3 Identification of differentially expressed genes (DEGs)

To find the DEGs between the control and experimental groups, the gene expression patterns of each group were normalized and analyzed using the “limma” package. The filtering criteria for the DEGs were set to a corrected p-value of < 0.05, |logFC| ≥ 1. A heatmap was visualized using the “pheatmap” package.

### 2.4 Functional enrichment analysis

“ClusterProfiler,” “enrichplot,” and “org.Hs.eg.db” packages were used to analyze important functions and pathways of DEGs, including Gene Ontology (GO) and Kyoto Encyclopedia of Genes and Genomes (KEGG) (Qin et al., 2023). The reference genome file “c2.cp.kegg.Hs.symbols.gmt” was used for gene set enrichment analysis (GSEA) to understand the differences in pathways between control and experimental groups (Qin et al., 2023). All results were visualized by the “ggplot2” software package.

### 2.5 PPI and MCODE analysis

The DEGs were uploaded to the online website STRING (http://string-db.org), and the PPI was constructed with a medium confidence level of 0.400. The PPI was then beautified by applying the software Cytoscape_3.8.0. MCODE analysis is to find out the key sub-networks and genes based on the relationship of edges and nodes in a huge PPI network, which facilitates downstream analysis to screen out the key genes (Bader and Hogue, 2003). Thus, MCODE in Cytoscape was chosen to calculate the information of each node in the PPI to produce the final functional module. The parameters were set as follows: Degree Cutoff: 2, Node Score Cutoff: 0.2, K-Core: 2, Max. Depth from Seed: 100.

### 2.6 Three ML algorithms for screening feature genes

We use the LASSO, SVM-RFE and RF algorithms (Qin et al., 2023) to screen key genes in the above functional modules. The feature genes were first screened using the LASSO algorithm to obtain a “LASSO coefficient path” and a “LASSO regularization path” (also known as Lasso regression analysis cross-validation curve). The former shows the variation of feature coefficients for different values of the regularization parameter (λ) in the LASSO algorithm. The latter shows the model fitting effect for different values of λ in the LASSO algorithm. The results of this figure allow us to find an optimal value of λ that gives the best Lasso fit and minimizes the cross-validation error. The number of genes corresponding to the point with the smallest cross-validation error is the number of disease signature genes. Then SVM-RFE algorithm can obtain a graph of cross-validation accuracy and a graph of cross-validation error. The horizontal coordinates of the two graphs represent the number of feature genes, and the vertical coordinates, “10 X CV Accuracy” and “10 X CV Error,” represent the accuracy and error rate of the curve changes after 10-fold cross-validation, respectively. In the next RF algorithm, random forest trees were first constructed by setting the number of trees n_tree_ = 500, obtaining a random forest tree graph. Find the number of trees corresponding to the point with the smallest cross-validation error in the graph as the best tree value. And score the importance of the genes based on the best tree value so as to rank the genes and select the genes with gene importance greater than 1 for subsequent analysis. Finally, the intersection of the three algorithm screening results was taken and the Venn diagram was plotted using the “VennDiagram” R package. The R package “pROC” and “InpROC” were also applied to plot the ROC curves and calculate the area under the curve (AUC), respectively, to determine the predictive value of these characterized genes in the training set and validation set.

### 2.7 Creation of ACAS nomogram

The R package “rms” “rmda” was applied to construct nomogram of the identified signature genes and a calibration curve was plotted to assess the accuracy of the nomogram. Then the clinical impact curves of the model were plotted and evaluated. Finally, the decision curve analysis was used to evaluate the clinical utility of the nomogram.

### 2.8 Immune-related functions and immune cell correlation analysis

The 59 ACAS samples in the training group were categorized into high and low groups according to the expression of target genes. The cited R packages “GSVA,” “GSEABase,” “ggpubr,” “reshape2,” and “ggExtra” show the differences of different immune-related functions between the high and low expression groups of the characterized genes, as well as explore the correlation analysis of the characterized genes with immune cells.

### 2.9 Immunohistochemistry and immunofluorescence double-labeling

Twenty specimens were first paraffin-embedded and then sliced into 5 µm thin slices using a paraffin slicer (Leica RM2235). Immunohistochemistry steps: Briefly, the sections were first dewaxed to water. Then antigen repair was performed under the condition (citric acid solution, microwave medium heat for 8 min, cease-fire for 8 min, turn to medium-low heat for 7 min). Endogenous peroxidase was next blocked with a 3% hydrogen peroxide solution. The tissue was covered evenly with drops of 3% BSA in the histochemistry circle and closed at room temperature for 30 min. Primary antibodies (C3AR1, GTX114293, 1:200, GeneTex; MPO, GB12224, 1:1500, Servicebio; MCP7, GB12110, 1; 500, Servicebio) were added and incubated overnight at 4°C. After cleaning, the slices were incubated with homologous secondary antibodies for another 1 h. Freshly prepared DAB solution was added to develop the color and then restained with hematoxylin for about 3 min. Then, the sections were dehydrated and sealed with xylene. Photographs were taken utilizing a microscope (Olympus BX53) and quantified using ImageJ (v1.8.0) analysis software.

Steps of homologous immunofluorescence double-labeling staining: the preparation process of the paraffin section, including antigen repair, was consistent with that of immunohistochemistry. The slices were added with the first primary antibody (C3AR1, GTX114293, 1:200, GeneTex) and incubated overnight at 4°C. After washing, incubate with the secondary antibody for 1 h. Then TSA dye was added and incubated for 10 min at room temperature away from light. After washing, antigen repair was again performed. The second primary antibody (MPO, GB12224, 1:4000, Servicebio) was supplemented and incubated overnight at 4°C and then incubated with the secondary antibody for another 1 h. Next, the nuclei were restained with DAPI and incubated at room temperature away from light for 10 min. Finally, images were captured using a fluorescence microscope (Olympus BX53) and quantified utilizing ImageJ (v1.8.0).

### 2.10 Construction of ceRNA and TF regulatory networks

The software miRanda, miRDB and TargetScan were applied to jointly predict miRNAs bound by characterized genes. miRNAs identified by all three software were saved for subsequent analysis. The spongeScan (Furió-Tarí et al., 2016) network was applied to predict miRNA-bound lncRNAs. The results were then imported into Cytoscape software to map the ceRNA regulatory network. Meanwhile, NetworkAnalyst (Zhou et al., 2019) (http://www.networkanalyst.ca) was utilized to construct the characteristic gene TFs regulatory network.

### 2.11 Statistical analysis

Statistical analysis was done using R version 4.2.3. The *t*-test was used for normally distributed variables and the Wilcoxon test was used for non-normally distributed variables. Linear relationships were analyzed using Pearson analysis, while monotonic relationships were analyzed using Spearman analysis. All statistical *p*-values were two-sided and *p* < 0.05 was considered statistically significant.

## 3 Results

### 3.1 177 DEGs were obtained

**FIGURE 1:**
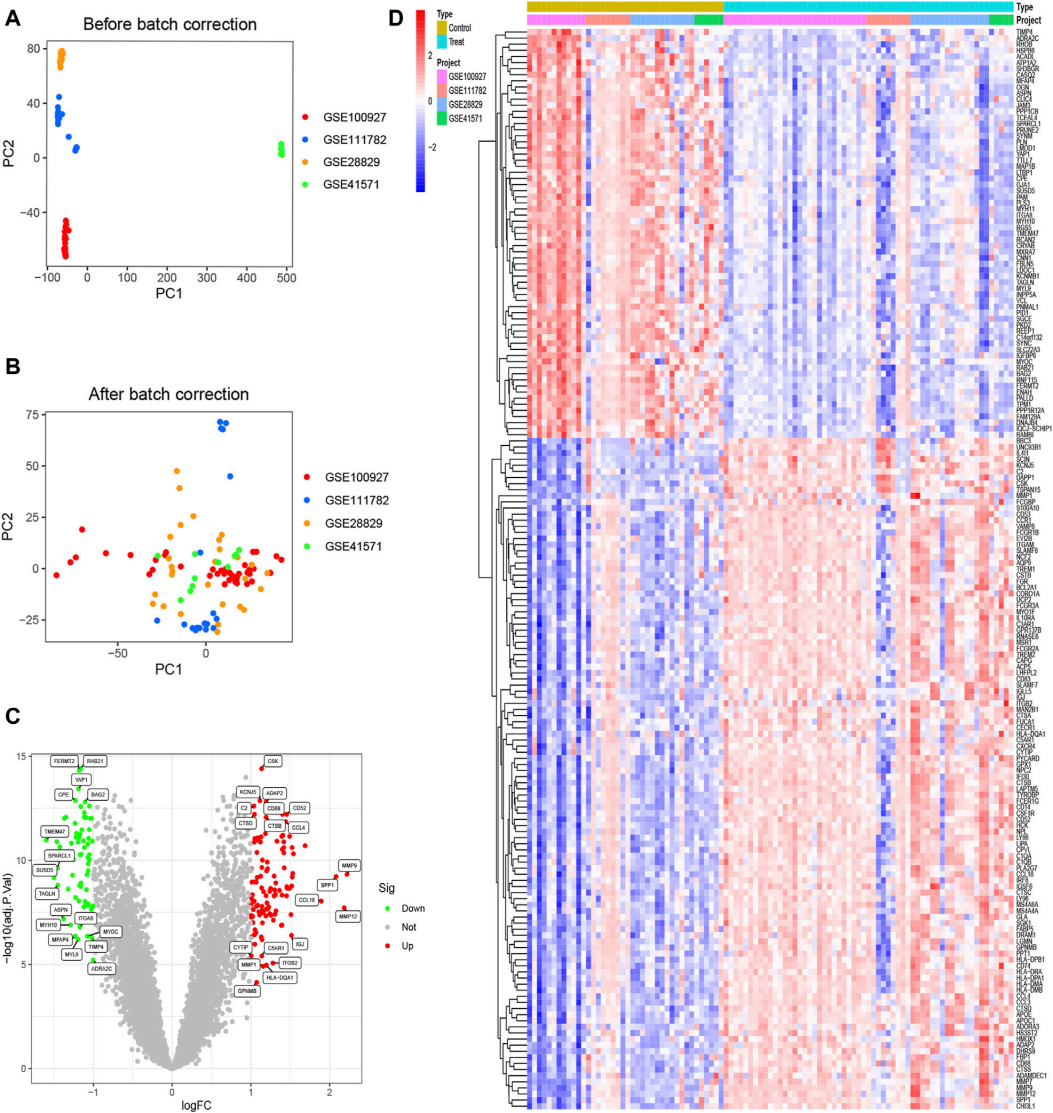
Identification of DEGs. **(A)** Samples from four datasets were shown to exist with batch effects; **(B)** Samples from four datasets eliminated the effects of batch effects; **(C)** Volcano plots of all genes; **(D)** Heatmaps of 67 downregulated genes and 110 upregulated genes.

Before performing the analysis of variance, we performed a batch correction. The PCA analysis showed that in the pre-correction graphs, the samples from different experiments were separated, meaning that there was a batch effect between these samples (Figure 1A). After batch correction, these samples were randomly distributed, eliminating the effect of batch effects (Figure 1B). All gene volcanoes were then mapped (Figure 1C). The final differential analysis yielded 177 DEGs, which contained 67 downregulated and 110 upregulated genes (Figure 1D).

### 3.2 Function and pathway exploration of 177 DEGs

**FIGURE 2:**
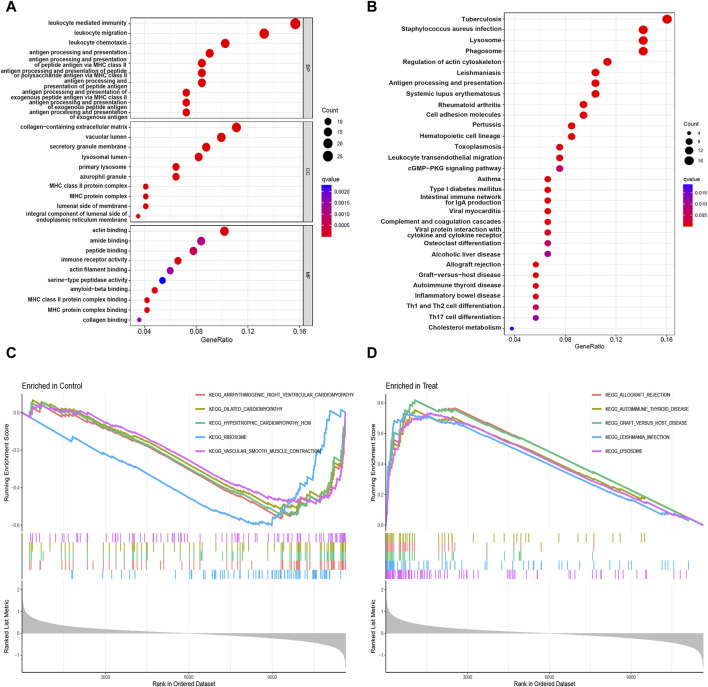
Functional enrichment analysis of 177 DEGs. **(A)** GO analysis results; **(B)** KEGG analysis results; **(C)** Five active pathways in the control group; **(D)** Five active pathways in the experimental group.

Next, functional enrichment analysis was performed on these DEGs. The GO and KEGG results indicated that these genes were primarily involved in leukocyte-mediated immunity, leukocyte migration, collagen-containing extracellular matrix, and actin binding functions (Figure 2A), as well as tuberculosis, *staphylococcus aureus* infection, lysosome, and phagosome pathways (Figure 2B). To understand the differences in pathways between the control and experimental groups, GSEA analysis was performed. The data demonstrated that these five pathways were active in the control: arrhythmogenic right ventricular cardiomyopathy, dilated cardiomyopathy, hypertrophic cardiomyopathy, ribosome, and vascular smooth muscle contraction (Figure 2C). In contrast, the experimental group showed activity in five different pathways: allograft rejection, autoimmune thyroid disease, graft versus host disease, leishmania infection, and lysosome (Figure 2D).

### 3.3 MCODE analysis yielded 7 important functional modules containing 63 genes

**FIGURE 3:**
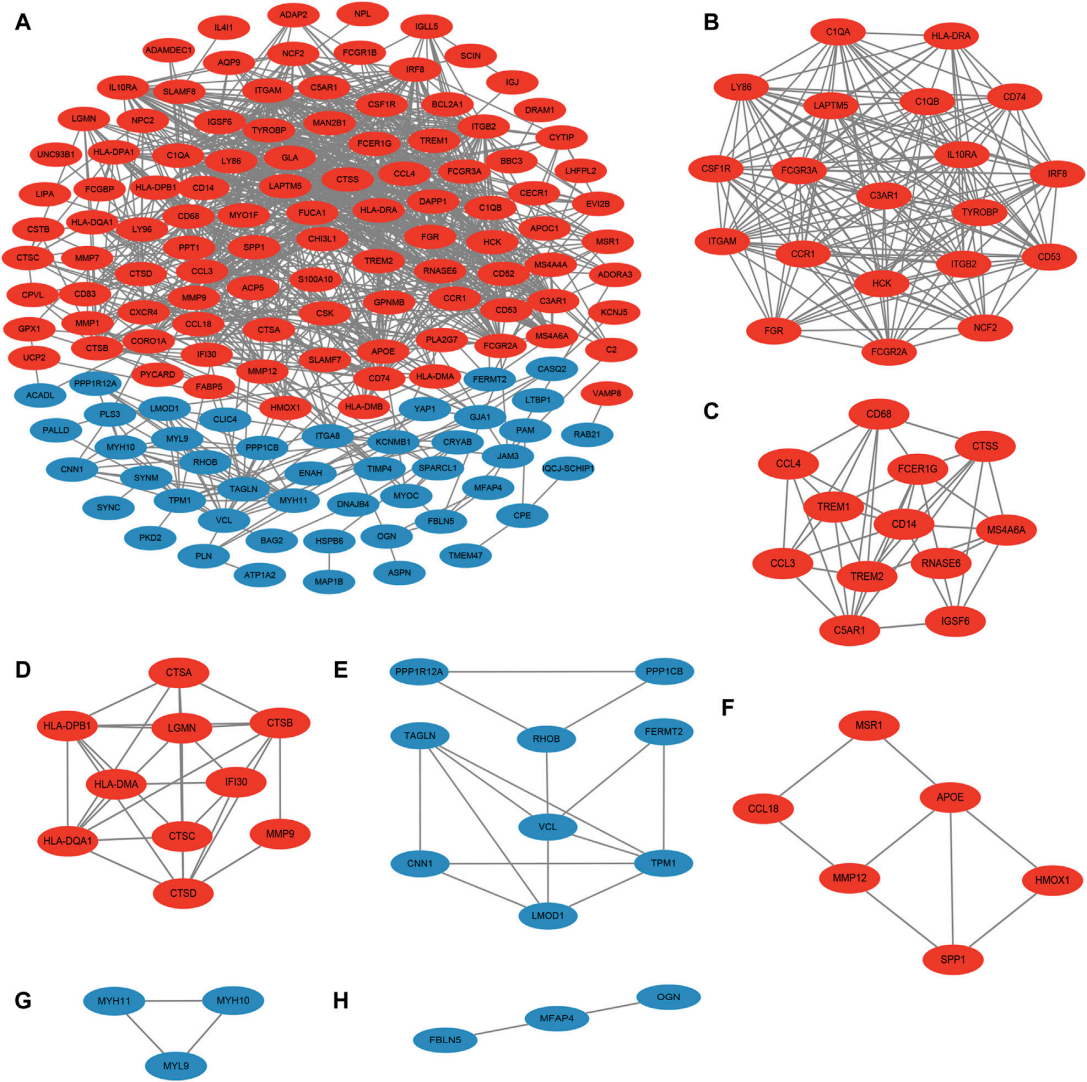
PPI and MCODE analysis. **(A)** PPI of DEGs; **(B–H)** seven important functional modules obtained from MCODE analysis. Red represents upregulated genes and blue represents downregulated genes.

Next, the PPI map of the 177 DEGs was constructed (Figure 3A). To further investigate the underlying mechanisms of ACAS, a modular network was created applying the MCODE algorithm to reveal the core therapeutic targets. The algorithm identified highly relevant network targets from the PPI network, and a total of 7 significant modules were generated (Figures 3B–H), containing 63 genes. Table 1 provides specific information for each module.

**TABLE 1:** The results of the MCODE analysis.

Cluster	Score	Nodes	Edges	Node IDs
1	17.895	20	170	LAPTM5, LY86, ITGB2, CD74, CD53, FCGR2A, IRF8, TYROBP, CSF1R, ITGAM, HLA-DRA, CCR1, C1QA, IL10RA, HCK, FCGR3A, FGR, NCF2, C1QB, C3AR1
2	7.091	12	39	CCL4, IGSF6, CD14, TREM1, CD68, CCL3, FCER1G, MS4A6A, RNASE6, CTSS, C5AR1, TREM2
3	6.444	10	29	IFI30, CTSA, CTSD, CTSC, HLA-DPB1, HLA-DQA1, HLA-DMA, MMP9, LGMN, CTSB
4	3.75	9	15	RHOB, LMOD1, PPP1CB, PPP1R12A, TPM1, TAGLN, CNN1, FERMT2, VCL
5	3.2	6	8	APOE, MMP12, CCL18, MSR1, HMOX1, SPP1
6	3	3	3	MYH11, MYH10, MYL9
7	3	3	3	FBLN5, OGN, MFAP4

### 3.4 Three ML algorithms screened for four feature genes

**FIGURE 4:**
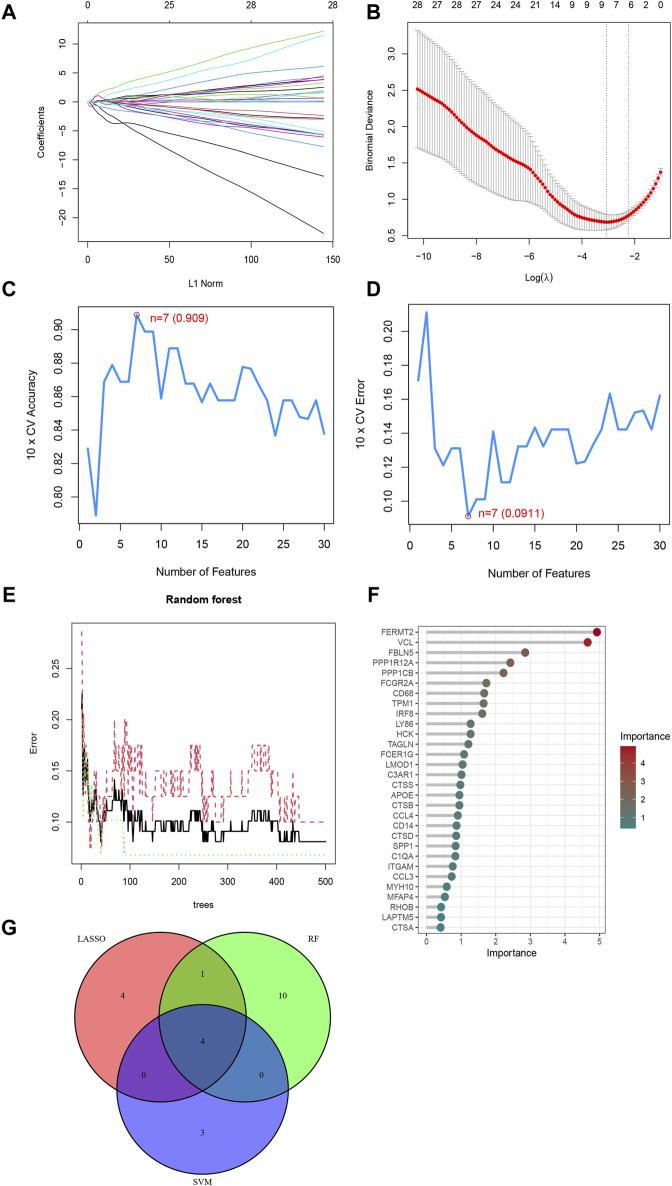
Three ML algorithms to screen feature genes. **(A)** LASSO coefficient path diagram, each curve represents one gene; **(B)** Lasso regression analysis cross-validation curve. When nine genes are used in the analysis, Lasso fits best and cross-validation error is minimized. **(C)** SVM-RFE algorithm determined the highest accuracy (0.909) when there were 7 genes; **(D)** SVM-RFE algorithm determined the lowest error rate (0.0911) when there were 7 genes; **(E)** The relationship between the number of Random Forest Trees and the error rate; **(F)** Genes are arranged in descending order of importance; **(G)** Venn diagrams of the genes obtained by the three algorithms.

Next, the study began with a LASSO analysis of 63 genes, resulting in the identification of 9 genes: C3AR1, CTSB, CTSD, FBLN5, FERMT2, MMP9, PPP1R12A, RHOB, and TPM1 (Figures 4A, B). Subsequently, the SVM-RFE algorithm was employed to screen seven genes, namely PPP1R12A, FBLN5, C3AR1, MYL9, HMOX1, MFAP4, and TPM1 (Figures 4C, D). Meanwhile, the RF algorithm identified 15 feature genes with relative importance greater than 1, including FERMT2, VCL, FBLN5, PPP1R12A, PPP1CB, FCGR2A, CD68, TPM1, IRF8, LY86, HCK, TAGLN, FCER1G, LMOD1, and C3AR1 (Figures 4E, F). Finally, we took the intersection of the genes screened by the three algorithms resulted in the identification of four characterized genes: C3AR1, FBLN5, PPP1R12A, and TPM1 (Figure 4G).

### 3.5 The four characterized genes had group differences in the control and experimental group

**FIGURE 5:**
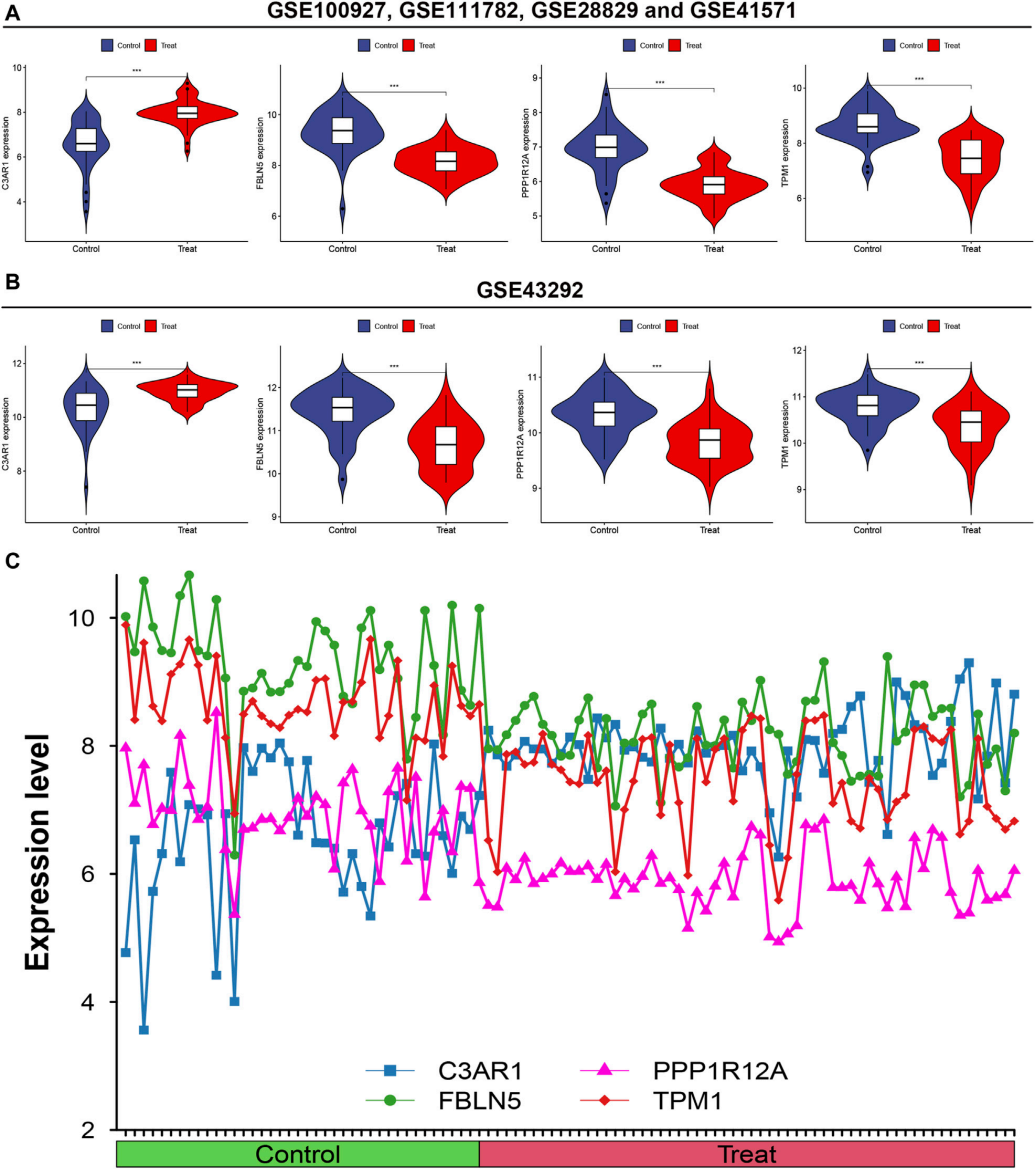
Intergroup difference analysis of the four characterized genes. **(A)** Differential analysis of the expression of the four feature genes in the training set illustrated by Violin plots; **(B)** Differential analysis of the expression of the four feature genes in the validation set shown by Violin plots; **(C)** Line plots of the expression levels of the four feature genes.

Adjacently, to probe into whether the expression of the four characterized genes differed between the control and experimental groups, violin plots and line plots were plotted. The results from both the training and validation sets indicate that the four characterized genes were differentially expressed in both the control and experimental groups (*p* < 0.01, Figures 5A, B). Additionally, C3AR1 was highly expressed in the experimental group, while FBLN5, PPP1R12A, and TPM1 were expressed at low levels in the experimental group (Figure 5C).

### 3.6 ROC analysis of the four characterized genes

**FIGURE 6:**
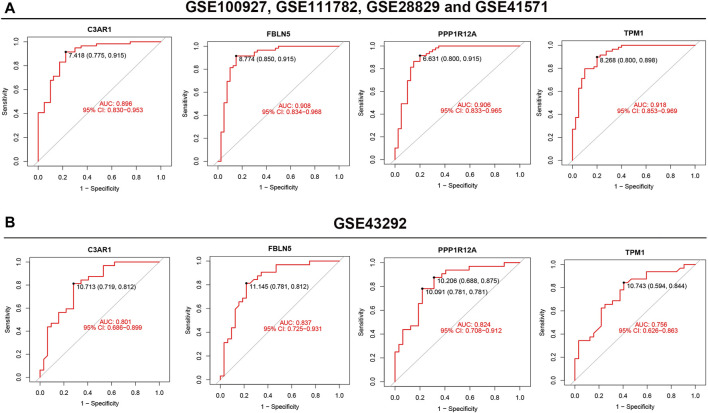
ROC analysis of the four feature genes. **(A,B)** ROC analysis results of the four feature genes in the training set **(A)** and validation set **(B)**, respectively.

ROC analysis was performed to verify the accuracy of the screened feature genes. In the training set, C3AR1, FBLN5, PPP1R12A and TPM1 had AUC values of 0.896, 0.908, 0.906, and 0.918, respectively (Figure 6A). In the validation set, the AUC values for C3AR1, FBLN5, PPP1R12A and TPM1 were 0.801, 0.837, 0.824, and 0.756, respectively (Figure 6B).

### 3.7 Construction of nomogram for predicting patients with ACAS based on four characterized genes

**FIGURE 7:**
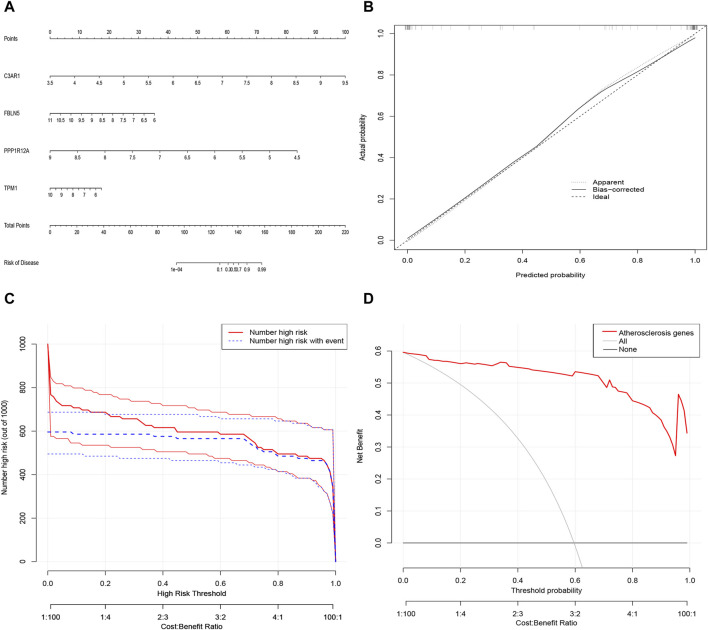
Alignment diagram model for predicting the risk of ACAS. **(A)** Alignment diagram for predicting ACAS. **(B)** Calibration curve to assess the predictive accuracy of the model. **(C)** Clinical impact curve to assess the model. **(D)** Decision curve analysis showing benefit in patients with ACAS.

Next, a nomogram was constructed as a diagnostic tool for ACAS by combining the four characterized genes (Figure 7A). The scores corresponding to each of the characterized genes were summed to obtain a total score, which corresponded to the risk of prevalence of ACAS. The calibration curve discovered that the accuracy of the nomogram in predicting prevalence was high (Figure 7B). The clinical impact curve also showed significant predictive power of the nomogram model (Figure 7C). Decision curve analysis hinted that patients with ACAS could benefit from the nomogram (Figure 7D).

### 3.8 Immune-related function and immune cell correlation analysis of the four characterized genes

**FIGURE 8:**
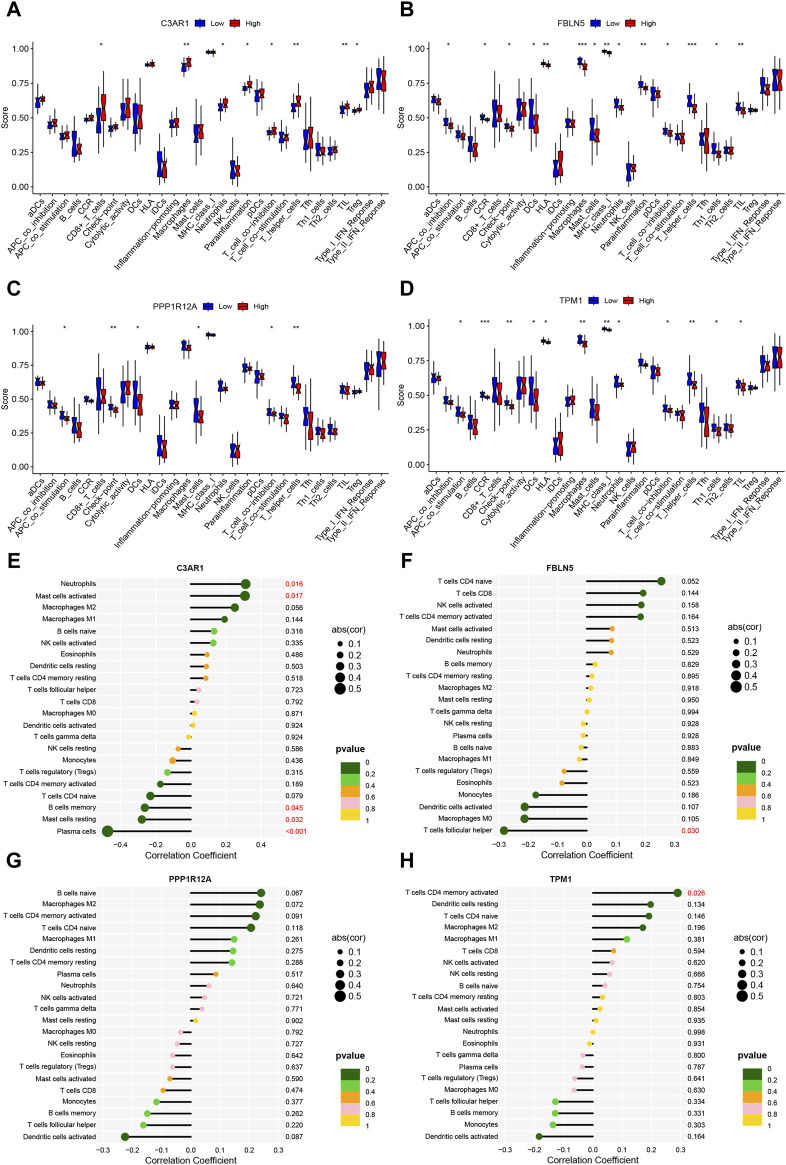
Immune-related functions and immune cell correlation analysis of characterized genes. **(A–D)** Box line plots of the differences between high and low expression groups for immune-related functions in C3AR1 **(A)**, FBLN5 **(B)**, PPP1R12A **(C)**, and TPM1 **(D)**, respectively; **(E–H)** Lollipop charts of the correlation of C3AR1 **(E)**, FBLN5 **(F)**, PPP1R12A **(G)** and TPM1 **(H)**, respectively, with 22 immune cell types.

Next, the immune-related function analysis displayed that diverse immune-related functions differed to varying degrees between high and low expression groups of the four characterized genes (Figures 8A–D). We then explored the correlation analysis between genes and immune cells. The results showed that C3AR1 expression was positively correlated with neutrophils and mast cells activated, and negatively correlated with B cells memory, mast cells resting, and plasma cells (Figure 8E). FBLN5 showed an inverse correlation with T cells follicular helper (Figure 8F), whereas TPM1 was positively correlated with T cells CD4 memory activated (Figure 8H). The statistical significance of PPP1R12A’s result was inconclusive (Figure 8G).

### 3.9 Immunohistochemical and fluorescent dual-labeling validation of C3AR1 expression in patients with ACAS

**FIGURE 9:**
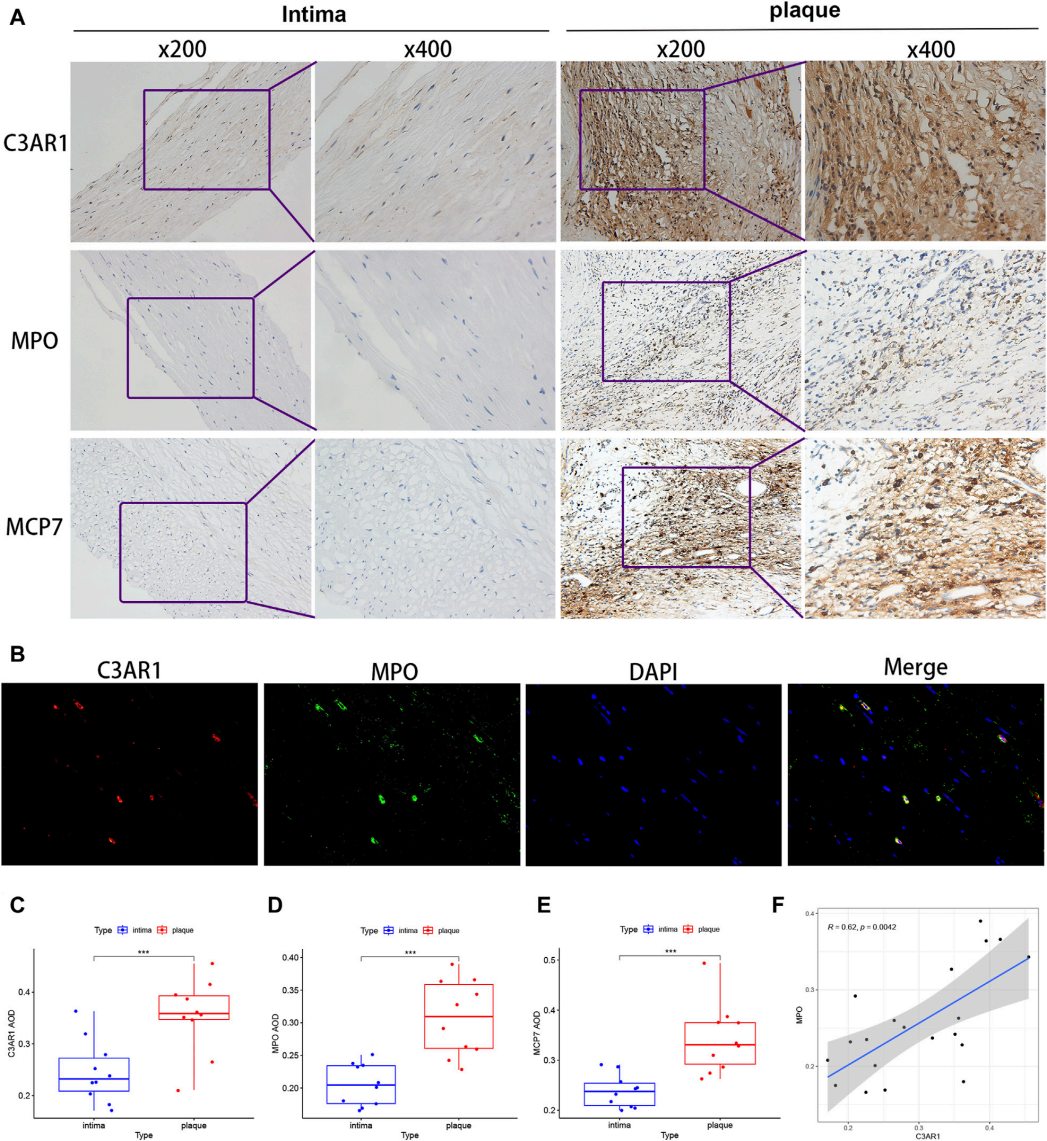
C3AR protein level in intima and plaques of patients with ACAS. **(A)** IH staining of C3AR1, MPO and MCP7 in the intima (left) and plaques (right) of patients with ACAS. **(B)** IF staining for C3AR1 (red) and MPO (green) in plaques from patients with ACAS (magnification, ×400). **(C–E)** Significant difference analysis of IH results for C3AR1, MPO and MCP7 present by box plots. ***, indicate *p* < 0.001. **(F)** Correlation plot of C3AR1 and MPO protein expression. MPO: myeloperoxidase (neutrophil marker); MCP7: mast cell protease 7 (mast cell marker).

Based on the results presented in Figure 8E, there appears to be a positive correlation between C3AR1 expression and neutrophils and mast cells activated in carotid atherosclerotic plaques. To verify this relationship, we examined the expression levels of C3AR1, myeloperoxidase (MPO), a neutrophil marker (Schmekel et al., 1990), and mast cell protease 7 (MCP7), a mast cell marker (Matsumoto et al., 1995), in the carotid intima and plaque tissues of various patients with ACAS who underwent CEA, using immunohistochemistry. Furthermore, through quantitative analysis, correlation analysis and immunofluorescence double labeling method, a close connection between C3AR1 and MPO as well as MCP7 was found in carotid atherosclerotic plaque tissues. More notably, IH staining revealed significantly higher expression of C3AR1, MPO, and MCP7 in plaques from patients with ACAS compared to the intima (*p* < 0.001, Figures 9A, C–E). IF double-labeling of plaques also revealed a significant co-localization relationship between C3AR1 and MPO-positive neutrophils (Figure 9B). Correlation analysis demonstrated a positive correlation between C3AR1 and both MPO expression level (Figure 9F) and MCP7 expression level (Supplementary Figure S1).

### 3.10 Construction of ceRNA and TFs regulatory networks for four characterized genes

**FIGURE 10:**
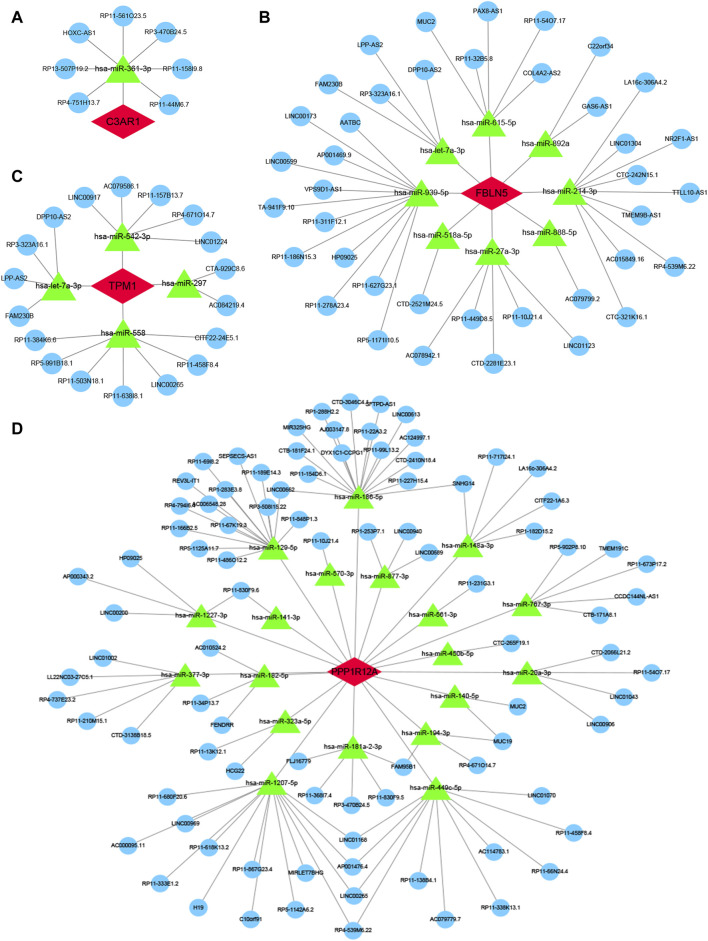
ceRNA of characterized genes. **(A–D)** The ceRNA regulatory networks of C3AR1 **(A)**, FBLN5 **(B)**, PPP1R12A **(C)**, and TPM1 **(D)**, separately. Red represents characterized genes, green represents miRNAs, and blue represents lncRNAs.

**FIGURE 11:**
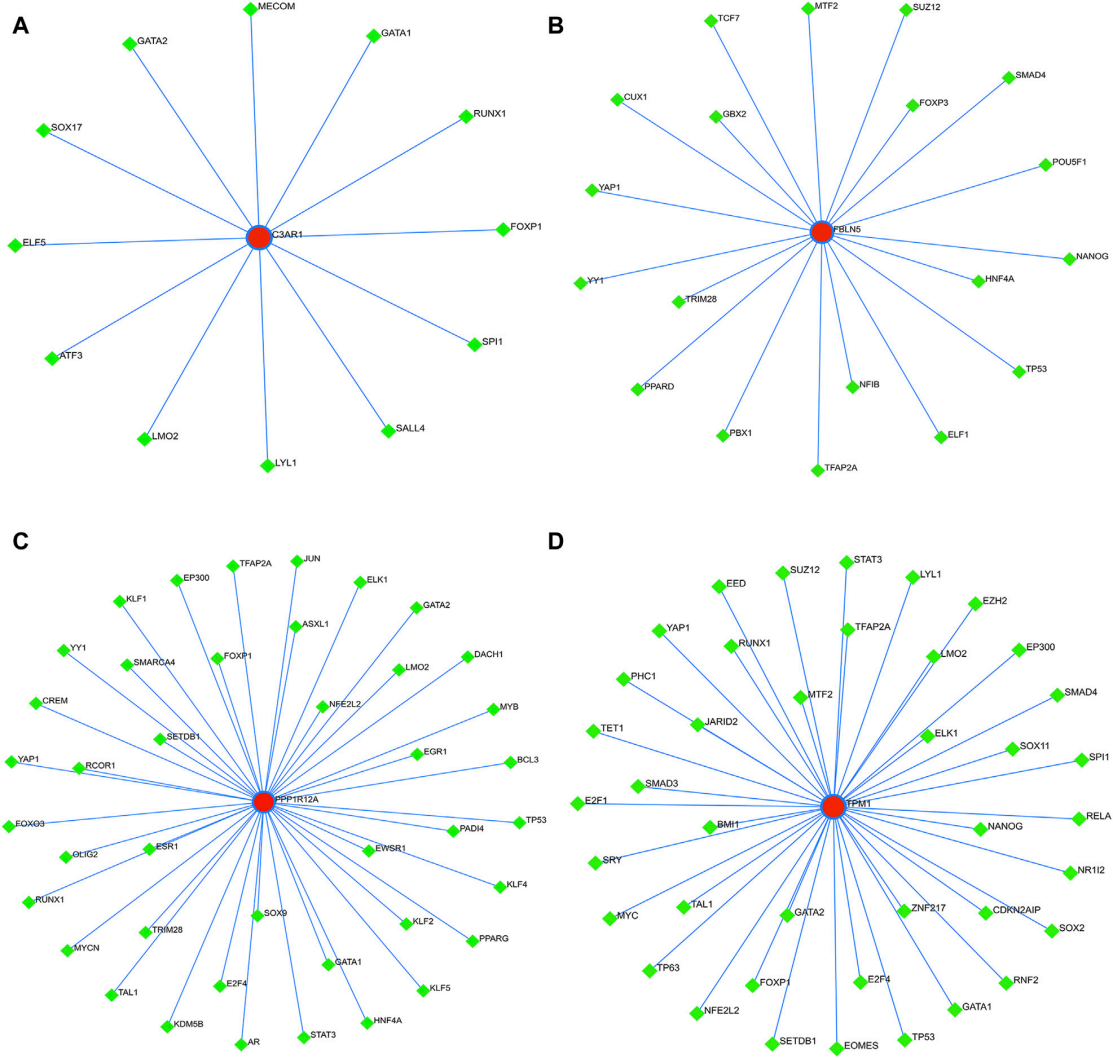
TFs regulatory networks of characterized genes. **(A–D)** TFs regulatory networks of C3AR1 **(A)**, FBLN5 **(B)**, PPP1R12A **(C)**, and TPM1 **(D)**, respectively.

Finally, to further explore the molecular mechanism of ACAS, the present study constructed the regulatory networks of ceRNA and TFs of four target genes. The ceRNA hypothesis reveals a new mechanism for RNA interactions. The ceRNA is a newly discovered mechanism to regulate gene expression, which includes mRNA encoding proteins, lncRNA, miRNA and circRNA (Salmena et al., 2011). We predicted the miRNAs bound to each characterized gene and also predicted the miRNA-bound lncRNAs. The results showed that seven lncRNAs competed with C3AR1 to bind hsa-miR-361-3p (Figure 10A). Thirty-nine lncRNAs competed with FBLN5 for binding to eight miRNAs (hsa-miR-27a-3p, hsa-miR-518a-5p, hsa-miR-939-5p, hsa-let-7a-3p, hsa-miR-888-5p, hsa-miR-615-5p, hsa-miR-892a, and hsa-miR-214-3p) (Figure 10B). Eighteen lncRNAs competed with TPM1 to bind four miRNAs (hsa-miR-542-3p, hsa-let-7a-3p, hsa-miR-558 and hsa-miR-297) (Figure 10C). While up to ninety-one lncRNAs competed with PPP1R12A for binding to nineteen miRNAs (hsa-miR-20a-3p, hsa-miR-450b-5p, hsa-miR-323a-5p, hsa-miR-767-3p, hsa-miR-148a-3p, hsa-miR-1207-5p hsa-miR-377-3p, hsa-miR-129-5p, hsa-miR-1227-3p, hsa-miR-561-3p, hsa-miR-182-5p, hsa-miR-141-3p, hsa-miR-181a-2-3p, hsa-miR-186-5p, hsa-miR-140-5p,hsa-miR-570-3p, hsa-miR-877-3p, hsa-miR-194-3p, and hsa-miR-449c-5p), respectively (Figure 10D). Thus, one or more lncRNAs would compete with the characterized genes to bind miRNAs. In addition, this study also predicted the TFs bound to each characterized gene. Among them, twelve transcription factors could bind to C3AR1 (Figure 11A). Nineteen transcription factors could bind to FBLN5 (Figure 11B). Forty-one transcription factors were able to bind to PPP1R12A (Figure 11C). And forty transcription factors were able to bind to TPM1 (Figure 11D). Thus, each characterized gene possesses multiple TFs.

## 4 Discussion

In recent years, due to hypertension, dyslipidaemia, diabetes, tobacco, obesity and other factors, cerebrovascular disease in young adults, especially ischemic stroke, has shown an increasing trend (Goldstein, 2020). Its extremely high mortality rate, disability rate, recurrence rate, and further complications bring a huge economic burden to people. Here, we investigated its main etiology, ACAS, and probed into the pivotal genes and regulatory networks associated with carotid atherosclerotic plaques employing bioinformatics methods.

Our study screened out four genes characterized by ACAS: C3AR1, FBLN5, PPP1R12A, and TPM1. Of these, C3AR1 was upregulated and FBLN5, PPP1R12A, and TPM1 were downregulated. The results of the analysis of variance in both the training and validation sets highlighted that the four characterized genes were differentially expressed in both the control and experimental groups. And the ROC analysis for the four genes revealed that they had high AUC values in both the training and validation sets, indicating the accuracy of our screening results. In addition, immunohistochemistry and fluorescence double labeling further confirmed that C3AR1 was highly expressed in atherosclerotic plaques of patients with ACAS. Therefore, we venture to hypothesize that these key diagnostic genes are tightly intertwined with the pathogenesis of ACAS and deserve to be explored in depth.

The C3AR1 gene encodes the C3a allergenic toxin chemotactic receptor, which belongs to the G protein-coupled receptor 1 family and stimulates chemotaxis, granzyme release and superoxide anion production. This gene is not only a key gene in carotid atherosclerosis (Meng et al., 2021), but also its signaling pathway C3a/C3aR1/VCAM1 mediates neuroinflammation in aging and neurodegenerative diseases (Propson et al., 2021). In our study, this gene also showed a positive correlation with the level of infiltrating neutrophils and mast cells activated. Previous studies have discovered that C3aR1 controls neutrophil mobilization after spinal cord injury through physiological antagonism of CXCR2 (Brennan et al., 2019). Besides, neutrophils can trigger atherosclerosis and promote atherosclerotic plaque destabilization and endothelial detachment (Silvestre-Roig et al., 2020). Notably, the formation of neutrophil extracellular traps (NETs) in neutrophils is one of the mechanisms of early atherosclerosis (Herrero-Cervera et al., 2022). Furthermore, activated diseased SMCs attract neutrophils to form NETs, which cause the histone H4 they contain to bind to and cleave SMCs, leading to plaque instability (Silvestre-Roig et al., 2019). Therefore, in our future studies, it is necessary to investigate how C3AR1 mediates the role of neutrophils in atherosclerosis and the potential specific mechanisms, so as to design neutrophil-targeted therapeutic strategies to stabilize atherosclerotic plaques, reverse ACAS, and reduce the incidence of stroke. In contrast, the interaction between C3AR1 and mast cells in atherosclerotic plaques has been less studied and needs to be explored in depth.

FBLN5 is a member of the fibronectin family and is essential for elastic fiber formation. It was discovered that FBLN5 may play an important role in carotid atherosclerosis via has-mir-128 and has-mir-532-3p (Zheng et al., 2022). PPP1R12A, also known as MYPT1, is a key regulator of protein phosphatase 1C. Evidence suggests that ROS-mediated downregulation of MYPT1 in smooth muscle cells is a potential mechanism for abnormal myocyte contractility in atherosclerosis (Cheng et al., 2013). TPM1, the pro-myosin α-1 chain, binds to actin filaments in muscle and non-muscle cells. It has been shown to be downregulated in unstable carotid atherosclerotic plaques (Guo et al., 2022). In short, these previous studies further support the reliability of our screening results. Hence, we established a predictive ACAS patient nomogram model based on the four characterized genes of our screening. This model can lead to the joint diagnosis or prediction of the pathogenic risk of patients with ACAS by the four characteristic gene indicators and provide an accurate digitalized risk probability for each patient, thus assisting clinicians in decision-making and individualized medical treatment.

Importantly, GSEA analysis revealed that five pathways were activated in the experimental group, encompassing xenograft rejection, autoimmune thyroid disease, graft-versus-host disease, leishmaniasis infection and lysosomes. It has been shown that allograft vasculopathy is a special case of immune-mediated atherosclerosis (Libby, 2012). Moreover, lysosomes are key nodes connecting lipid degradation, autophagy, apoptosis, inflammatory vesicles, lysosomal biogenesis and macrophage polarization, and may play a predominant role in the initiation, development and progression of atherosclerotic plaques (Zhang et al., 2021). However, the remaining pathways such as autoimmune thyroid disease, graft-versus-host disease and leishmaniasis infection have not been reported to be associated with atherosclerosis. Therefore, future exploration of the role of these pathways in ACAS may offer more effective and precise avenues for drug development and therapy.

More intriguingly, with the completion of the human genome sequencing project and the continuous optimization of sequencing technologies, the richness of the RNA world and the diversity of TFs have been continuously recognized, opening up new frontiers for the treatment of diseases. Therefore, to further enrich future therapeutic strategies for ACAS, we constructed the ceRNA and transcription factor regulatory networks of four target genes.

The ceRNA include mRNA, miRNA, lncRNA and so on (Salmena et al., 2011). Studies have shown that many miRNAs are involved not only in atherosclerosis-related physiological and pathological processes, but also in lipid processing, inflammation and cellular behaviors (such as proliferation, migration and phenotypic transformation) (Navarro et al., 2020). For example, extracellular vesicles-derived hsa-miR-27a-3p promotes M2 macrophage polarization, thereby promoting cell proliferation and migration (Zhao et al., 2022). Inhibition of hsa-miR-140-5p expression can induce upregulation of C-reactive protein, which is involved in atherogenesis (Teng and Meng, 2019). In addition, one or more lncRNAs compete with signature genes to bind miRNAs. lncRNAs coordinate and integrate a variety of signaling pathways and play important roles in development, differentiation and disease (Navarro et al., 2020). lncRNAs affect the expression levels of genes closely related to endothelial dysfunction, smooth muscle cell proliferation, macrophage dysfunction, abnormal lipid metabolism and cellular autophagy in atherosclerotic plaques, and thus are involved in regulating the onset and progression of atherogenesis (Ma et al., 2023). For example, the long non-coding RNA HOXC-AS1 inhibits oxidized low-density lipoprotein (ox-LDL)-induced cholesterol accumulation by promoting the expression of HOXC6 in THP-1 macrophages (Huang et al., 2016). LINC01123 is highly expressed in patients with CAS and promotes cell proliferation and migration by regulating the ox-LDL-induced miR-1277-5p/KLF5 axis in vascular smooth muscle cells (Weng et al., 2021). In addition, TFs can regulate macrophages in atherosclerosis through mechanisms involved in cytokine signaling, lipid signaling, and foam cell formation (Kuznetsova et al., 2020). For instance, decreasing RUNX1 expression in macrophages inhibits ox-LDL-induced lipid accumulation and inflammation (Liu et al., 2022). Endothelial Foxp1 inhibits atherosclerosis by regulating Nlrp3 inflammasome activation (Zhuang et al., 2019). In conclusion, these findings further support the accuracy of the ceRNA and TFs regulatory networks of the characterized genes constructed in this study. Therefore, an in-depth understanding of the mechanisms and functions of these ceRNA and TFs will help us to better ravel out the mysteries of the regulation of these characterized genes and provide new ideas for the future treatment of ACAS.

Undeniably, there are some limitations to this study. First, although this study identified four signature genes for ACAS based on ML algorithms and validated their diagnostic efficacy in an external dataset, prospective cohorts are needed to further investigate the biological significance of these signature genes in predicting ACAS. Second, we validated the high expression of C3AR1 in patients with ACAS only in human plaque tissue, whereas the levels of FBLN5, PPP1R12A and TPM1 in plaques need to be further clarified. In conclusion, further *in vivo* and *in vitro* studies are needed to elucidate the potential mechanisms of action of C3AR1, FBLN5, PPP1R12A and TPM1 in ACAS.

## 5 Conclusion

Four pivotal genes were screened, and relevant ceRNA and TFs were predicted. These molecules may play a crucial role in ACAS and serve as potential biomarkers and therapeutic targets.

## Data Availability

The original contributions presented in the study are included in the article/Supplementary Material, further inquiries can be directed to the corresponding author.

## Abbreviations

ACAS, atherosclerotic carotid artery stenosis; AUC, area under the curve; CAS, carotid artery stenosis; ceRNA, competing endogenous RNAs; DEGs, differentially expressed genes; GO, Gene Ontology; GSEA, gene set enrichment analysis; KEGG, Kyoto Encyclopedia of Genes and Genomes; LASSO, least absolute shrinkage and selection operator; MCODE, molecular complex detection; ML, machine learning; MCP7, mast cell protease 7; MPO, myeloperoxidase; NETs, neutrophil extracellular traps; PCA, principal component analysis; PPI, Protein-protein interaction networks; RF, random forest; SVM-RFE, support vector machine-recursive feature elimination; TF, transcription factor; TIA, transient ischemic attack.
