# Supplementary figures and images for: Identification of pivotal genes and regulatory networks associated with atherosclerotic carotid artery stenosis based on comprehensive bioinformatics analysis and machine learning
Source: Front Pharmacol. 2024 Apr 17;15:1364160. doi: 10.3389/fphar.2024.1364160 (PMC11061441; doi:10.3389/fphar.2024.1364160)

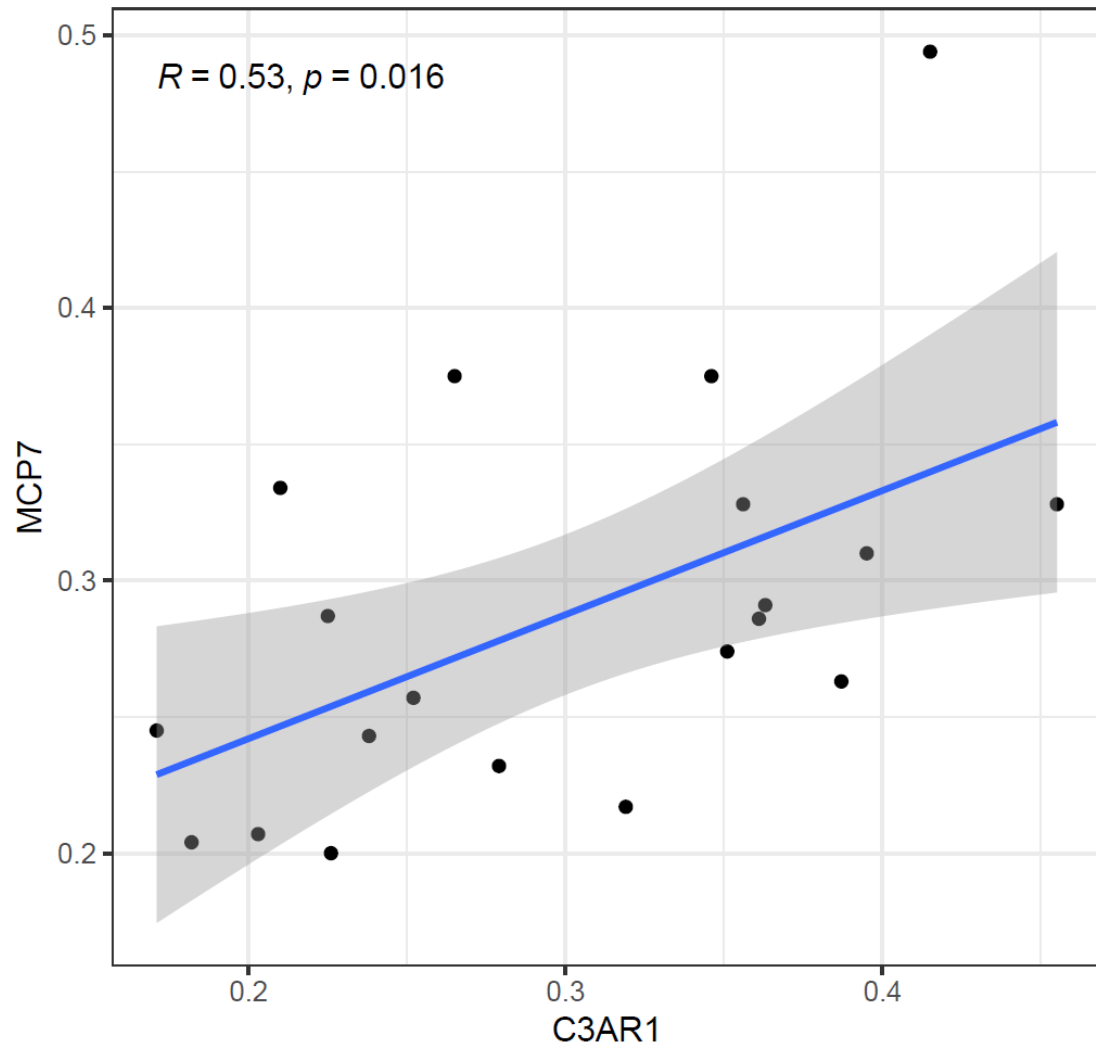

**Fig. S1** Correlation plot of C3AR1 and MCP7 expression.

Supplement: Supplementary file 1 [file Image1.pdf]
